# Therapy of ocular complications in ANCA+ associated vasculitis

**DOI:** 10.22336/rjo.2021.3

**Published:** 2021

**Authors:** Sorin Simion Macarie, Răzvan-Marian Mihăilă, Daniela Mariana Macarie, Dan-Alexandru Toc

**Affiliations:** * “Iuliu Hațieganu” University of Medicine and Pharmacy, Cluj-Napoca, Romania; ** Cluj County Emergency Hospital, Cluj-Napoca, Romania; ** A.I. S.C.B.I. Cluj, Cluj-Napoca, Romania

**Keywords:** vasculitis, ocular, orbital

## Abstract

ANCA+ associated vasculitis (AAV) is a group of rare diseases with potentially vision-threatening complications. Ocular and orbital complications of these diseases are caused by vasculitis of the small vessels of the eye or by granulomatous mass formation. ANCA (anti-neutrophil cytoplasmic antibodies) represent a key component of pathophysiological pathways as well as a diagnostic marker. Various manifestations are reported in literature, scleritis and episcleritis being the most common, followed by pseudotumor orbitae. In vision-threatening orbital or ocular disease, aggressive systemic treatment with a combination of high-dose glucocorticoids and either cyclophosphamide or rituximab is needed. Certain cases require locoregional surgical management to preserve ocular integrity or vision. Ocular involvement of AAV remains a challenge in clinical practice, requiring multi-specialty cooperation in order to ensure the best possible visual outcome.

**Abbreviations:** AAV = ANCA+ associated vasculitis, ANCA = anti-neutrophil cytoplasmic antibodies, GPA = granulomatosis with polyangiitis, EGPA = eosinophilic granulomatosis with polyangiitis, MPA = microscopic polyangiitis

## Introduction

Vasculitis is a heterogenous group of rare systemic diseases with unclear causes that are characterized by inflammatory cellular infiltrates with or without necrosis in the vessel walls. ANCA (anti-neutrophil cytoplasmic antibodies) are the hallmark of three small-vessel vasculitis, namely granulomatosis with polyangiitis (GPA), eosinophilic granulomatosis with polyangiitis (EGPA) and microscopic polyangiitis (MPA) [**1**]. These three conditions are known and studied together as ANCA negative associated vasculitis (AAV). The combined incidence of AAV vasculitis is approximately 20 cases per million in the general population. Among the three types, EGPA has the lowest incidence, with each of the other two types occupying the first place depending on geographical and ethnical factors [**2**]. There is a slight male predominance. AAV predominantly occurs between the ages of 35 and 55, but it can occur at any age, including in rare pediatric cases.

Apart from the potentially life-threatening systemic complications of the disease, patients can also present ocular and/ or orbital manifestations, some of them serious and vision-threatening. A retrospective study of 1286 patients with necrotizing vasculitis showed that up to one half of the patients with GPA present some form of ocular involvement, which can sometimes be the inaugural presentation of the disease. Ocular complications occur much less frequently in EGPA and MPA, with reported findings of less than 10% of the patients [**3**].

When patients affected by AAV were asked about the subjective impact the disease has on their lives, a Mexican study [**4**] showed that almost 20% stated that visual abnormalities are an important aspect of their disease. We can safely conclude that ophthalmologists play an important role in the management of AAV-related ocular complications and they should be prepared in case they encounter this rare, but possibly vision impairing disease.

In order to be able to understand the treatment of ocular involvement in AAV, it is necessary that its pathogenesis and clinical presentation is shortly explained.

## Pathophysiology of ocular involvement

The etiology of AAV is currently unknown, although there is evidence of environmental risk factors based on the geographical disparities of disease incidence in the world. These factors act on a genetical predisposition that is currently poorly defined.

Two immunological pathways are described. The first one is the neutrophil pathway: ANCA bind to membrane-bound myeloperoxidase (MPO) and proteinase-3 (PR-3) molecules of neutrophils, causing them to adhere to the endothelial wall of small vessels and degranulate, resulting mainly in the necrotizing vasculitis component of the disease. Thus, it is important to note that ANCA are not only a marker of AAV, but also have a crucial role in its pathogenesis. The second pathway involves T cells: it was demonstrated that the upregulation of effector memory T cells and downregulation of regulatory T cells lead mainly to granulomatous inflammation of tissues and promote the ANCA production via T cell-B cell interaction [**5**].

While GPA and EGPA cause granulomatous inflammation in tissues, MPA is not known to cause such inflammation.

Although the eye is considered an immune privileged site, the vasculitis can affect any of the scleral, episcleral, and limbal vessels, as well as retinal and choroidal vessels, resulting in the loss of the natural barriers of the eye and subsequent inflammation.

Orbital inflammation usually translates into orbital granulomatous masses, often appearing as tumoral masses. The granulomas can develop from the retroorbital fat or, more commonly, through contiguity from the neighboring structures, specifically the paranasal sinuses, meninges, or lacrimal gland [**6**]. Orbital masses can appear as part of a systemic disease or can be described as a limited form of AAV.

## Ocular and orbital clinical presentation

The ocular manifestations in AAV can be very diverse, ranging from mild conditions, such as conjunctivitis, to vision-threatening diseases, such as scleritis and keratitis. All ocular symptoms should be immediately referred to an ophthalmologist, as many of the ocular presentations of a patient with AAV can fall under the “red eye” category, with very different prognoses.

The granulomatous inflammation can develop in the orbit, causing inflammatory pseudotumor. It develops in up to 30% of the patients with GPA, with few cases appearing in patients with EGPA and virtually none in the MPA arm of AAV [**3**]. The pseudotumor can cause proptosis, diplopia, orbital pain and optic nerve compression with visual acuity (VA) reduction. The evolution of the disease can be extremely severe, with vision loss and local pain that ultimately lead to enucleation [**7**]. There are case reports of granulomas appearing within the vascularized structures of the eye, mainly the choroid, although these remain extremely rare [**8**,**9**].

Vasculitis of the conjunctival, episcleral, scleral, limbal and uveal vessels can cause inflammation of their respective structures of the eye. In GPA, episcleritis and scleritis are prominent findings among patients, with uveitis and keratitis manifesting more rarely [**3**]. These ocular findings can be isolated but are often found as associations. To our knowledge, there is no study that systematically describes the characteristics of keratitis in AAV. However, according to multiple case reports, it occurs primarily in the form of peripheral ulcerative keratitis [**10**-**12**].

Dry eye syndrome is reported to occur in patients with AAV [**12**], although it is not a specific finding, as the reported prevalence of dry eye ranges from 5% up to 50% in the general population [**13**]. On the other hand, epiphora is one of the most common symptoms reported by patients, which is caused by the obstruction of the lacrimal drainage system by inflammation and nasal involvement [**14**].

Rare reports of retinal and choroidal vasculitis have been presented, most of them appearing in GPA patients [**15**]. Similarly, retinal vessel occlusions have been reported in rare instances [**16**,**17**].

## Work-up and diagnosis of AAV

A diagnosis of AAV is usually difficult to establish, as symptoms tend to be non-specific. Nonetheless, a multisystemic disease imposes a high level of suspicion.

Laboratory work-up and serologic tests should be performed. ANCA testing remains an important pillar in the decision-making process regarding the diagnosis and treatment of AAV. Still, a low probability of AAV diagnosis will yield a low post-test probability of the disease [**18**]. Therefore, ANCA immunoassays should be ordered only in high probability clinical situations. Different types and targets of ANCA can offer more information on the type of AAV and its characteristics.

Imagistic explorations of chest, brain, orbit, ear, nose, and throat are required in order to complete the clinical assessment of the patient.

A biopsy of the affected organ should be performed every time it is possible in order to confirm the specific alterations of the necrotizing vasculitis in the specimens and help establish the diagnosis [**19**], although the lack of biopsiable tissue should not delay the initiation of treatment [**1**].

## Treatment of ocular complications in AAV

Regarding the therapy of ANCA-associated vasculitis, the most recently updated comprehensive guideline was developed in 2008 by an international task force coordinated by EULAR (European League against Rheumatism) [**20**]. EULAR, together with ERA (European Renal Association) and EUVAS (European Vasculitis Society), published the latest update in 2016. Although this particular guideline does not explain the course of action in case of ocular complications, the Canadian Vasculitis research network (CanVasc) defines severe ANCA-associated vasculitis as any lesion that represents a major organ-threatening condition, including eye involvement [**21**]. Thus, the therapy recommendations in the case of severe disease can be extrapolated to the ophthalmological field.

In case of organ-threatening disease, the course of action recommended by the EULAR guideline for obtaining a remission is a combination of a high-dose systemic corticosteroid and either cyclophosphamide or rituximab. After achieving remission, cyclophosphamide must be discontinued due to its severe long-term side effects. Treatment will be continued with another maintenance drug (immunomodulator e.g., azathioprine, methotrexate, or biological therapy such as rituximab), along with continued corticosteroid tapering. Absence of remission should be managed by switching to rituximab if using cyclophosphamide or vice versa. In addition, the refractory cases should be referred to expert centers and possibly included in clinical trials. Relapses should be treated with the previously mentioned remission-inducing agents, as if dealing with a newly presented disease [**19**]. These are the high-level recommendations for GPA and MPA. There are no definite treatment recommendations for EGPA, because of its rarity and unique pathogenetic features, which led to some non-conclusive clinical trials [**22**]. Traditionally, EGPA flares are managed using high-dose corticosteroids. Despite the limited evidence, immunomodulators are used to induce and maintain remission of cortico-resistant EGPA with ocular involvement and some success [**23**]. Newer therapy agents, including rituximab, mepolizumab and omalizumab, were used in EGPA, but to the authors’ knowledge, there has been no study or case report involving ocular presentation published to date.

The EULAR guideline can be applied in ophthalmological context, as studies and case reports showed efficacy in treating ocular inflammation. For instance, a small study [**24**] of 13 patients (19 eyes) suffering from scleritis, uveitis and/ or peripheral ulcerative keratitis proved that both cyclophosphamide and rituximab induced remission of the inflammation, with most of the treated eyes (79%) preserving or improving vision at follow-up. Another study [**25**] of 16 patients with GPA and peripheral ulcerative keratitis showed slightly better results in controlling inflammation and long-term relapse rate after treatment with rituximab than cyclophosphamide.

A case report [**10**] of GPA with scleritis showed signs of worsening and occurrence of peripheral ulcerative keratitis under treatment with cyclophosphamide, but induction of remission under rituximab proved that switching between the two main remission-inducing agents is beneficial in severe ocular involvement.

Scleral and corneal thinning may be severe enough to endanger the visual function through perforation or rupture, regardless of systemic medication, thus necessitating surgical management, such as conjunctival resections, scleral grafts, tissue gluing and patching, lamellar or penetrating keratoplasty or tarsorrhaphy [**25**,**26**].

Regarding the orbital masses that are found mainly in GPA patients, high-dose corticosteroids alone or, more often, associated with immunomodulators represent the first-line treatment. In two series of case reports [**6**,**27**], rituximab seemed to provide better results than cyclophosphamide. However, granulomatous masses remain difficult to treat, as there is a significant number of refractory or worsening cases, patients necessitating multiple lines of treatment, complications leading to visual impairment and loss of vision (72% and 19% respectively in a study [**28**] including 40 patients with GPA and orbital mass).

Surgical management of orbital masses is required in up to 50% of the cases, specifically those presenting persistent pain or progressive loss of vision despite the optimal medication. Orbitotomy with orbital decompression or orbital debulking surgery is the preferred approach, but in certain severe cases, with marked proptosis, blindness, and severe pain, exenteration may be required. Orbital corticosteroid injections can be administered. Distinction between active inflammatory masses and fibrous sequelae of previous active episodes is necessary, because both medical and surgical treatment show little or no benefit in chronic changes with fibrosis. Orbital fibrosis manifests with enophthalmos, eye movement limitation and loss of vision [**29**,**30**].

Non-vision-threatening inflammation of the eye (e.g. conjunctivitis, episcleritis) can be safely treated with topical corticosteroids only [**31**]. Dry eye syndrome has no particularity in AAV and is managed as per usual [**12**].

Regarding epiphora caused by nasolacrimal duct obstruction in GPA, external dacryocystorhinostomy with silicone tubing is the surgical technique of choice, with good results and few postoperative complications [**32**,**33**]. The prerequisite for surgical success is a good control of local inflammation using the previously mentioned medications.

## Conclusion

AAV is a group of rare diseases with potentially disastrous visual consequences. Ocular manifestations in AAV are the result of either small-vessel vasculitis or granulomatous inflammation. Ocular and orbital inflammation can take many forms, but the most common remain episcleritis/ scleritis and orbital granuloma mass. A high degree of suspicion must be maintained and prompt liaison with rheumatologists must be established in order to initiate treatment in proper time. Often, local surgical management is necessary to maintain ocular integrity or vision.

**Conflicts of interest**

None.

**Acknowledgements**

None.

**Sources of funding**

None.

**Disclosures**

None.

## References

[1] Yates M, Watts R (2017). ANCA-associated vasculitis. Clin Med (Northfield Il).

[2] Watts R, Robson J (2018). Introduction, epidemiology, and classification of vasculitis. Best Pract Res Clin Rheumatol.

[3] Rothschild P-R, Pagnoux C, Seror R, Brezin AP, Delair E, Guillevin L (2013). Ophthalmologic Manifestations of Systemic Necrotizing Vasculitides at Diagnosis: A Retrospective Study of 1286 Patients and Review of the Literature. Semin Arthritis Rheum.

[4] Hinojosa-Azaola A, Jiménez-González A, Alcocer-Castillejos N (2018). Patient and physician perspectives on the impact of health-related quality of life in Mexican patients with ANCA-associated vasculitis. Rheumatol Int.

[5] Wilde B, Van Paassen P, Witzke O, Tervaert JWC (2011). New pathophysiological insights and treatment of ANCA-associated vasculitis. Kidney Int.

[6] Durel C, Hot A, Trefond L, Aumaitre O, Pugnet G, Samson M (2019). Orbital mass in ANCA-associated vasculitides: data on clinical, biological, radiological and histological presentation, therapeutic management, and outcome from 59 patients. Rheumatology.

[7] Ismailova DS, Abramova JV, Novikov PI, Grusha YO (2018). Clinical features of different orbital manifestations of granulomatosis with polyangiitis. Graefe’s Arch Clin Exp Ophthalmol.

[8] Levy-Clarke G, Ding X, Gangaputra S, Yeh S, Goodglick T, Byrnes G (2009). Recalcitrant granulomatous sclerouveitis in a patient with granulomatous ANCA-associated vasculitis. Ocul Immunol Inflamm.

[9] Proia AD (2011). Granulomatous Choroiditis in Wegener Granulomatosis. Arch Ophthalmol.

[10] Fujita Y, Fukui S, Endo Y, Tsuji S, Takatani A, Shimizu T (2018). Peripheral ulcerative keratitis associated with granulomatosis with polyangiitis emerging despite cyclophosphamide, successfully treated with rituximab. Intern Med.

[11] Gu J, Zhou S, Ding R, Aizezi W, Jiang A, Chen J (2013). Necrotizing Scleritis and Peripheral Ulcerative Keratitis Associated with Wegener’s Granulomatosis. Ophthalmol Ther.

[12] Schmidt J, Pulido JS, Matteson EL (2011). Ocular manifestations of systemic disease: Antineutrophil cytoplasmic antibody-associated vasculitis. Curr Opin Ophthalmol.

[13] Stapleton F, Alves M, Bunya VY, Jalbert I, Lekhanont K, Malet F (2017). TFOS DEWS II Epidemiology Report. Ocul Surf.

[14] Hinojosa-Azaola A, García-Castro A, Juárez-Flores A, Recillas-Gispert C (2019). Clinical significance of ocular manifestations in granulomatosis with polyangiitis: association with sinonasal involvement and damage. Rheumatol Int.

[15] Lin M, Anesi SD, Ma L, Ahmed A, Small K, Foster CS (2018). Characteristics and Visual Outcome of Refractory Retinal Vasculitis Associated With Antineutrophil Cytoplasm Antibody–Associated Vasculitides. Am J Ophthalmol.

[16] Yasude T, Kishida D, Tazawa K, Matsuda M, Ishii W, Yazaki M (2012). ANCA-associated vasculitis with central retinal artery occlusion developing during treatment with methimazole. Intern Med.

[17] De Salvo G, Li Calzi C, Anastasi M, Lodato G (2009). Branch retinal vein occlusion followed by central retinal artery occlusion in Churg-Strauss syndrome: Unusual ocular manifestations in allergic granulomatous angiitis. Eur J Ophthalmol.

[18] Csernok E (2013). ANCA testing: The current stage and perspectives. Clin Exp Nephrol.

[19] Yates M, Watts RA, Bajema IM, Cid MC, Crestani B, Hauser T (2016). EULAR/ERA-EDTA recommendations for the management of ANCA-associated vasculitis. Ann Rheum Dis.

[20] Geetha D, Jin Q, Scott J, Hruskova Z, Hanouneh M, Little MA (2018). Comparisons of Guidelines and Recommendations on Managing Antineutrophil Cytoplasmic Antibody–Associated Vasculitis. Kidney Int Reports.

[21] McGeoc L, Twilt M, Famorca L, Bakowsky V, Barra L, Benseler SM (2016). CanVasc recommendations for the management of antineutrophil cytoplasm antibody-associated vasculitides. J Rheumatol.

[22] Furuta S, Iwamoto T, Nakajima H (2019). Update on eosinophilic granulomatosis with polyangiitis. Allergol Int.

[23] Akella SS, Schlachter DM, Black EH, Barmettler A (2019). Ophthalmic Eosinophilic Granulomatosis With Polyangiitis (Churg-Strauss Syndrome): A Systematic Review of the Literature. Ophthal Plast Reconstr Surg.

[24] Ahmed A, Foster CS (2019). Cyclophosphamide or Rituximab Treatment of Scleritis and Uveitis for Patients with Granulomatosis with Polyangiitis. Ophthalmic Res.

[25] Ebrahimiadib N, Modjtahedi BS, Roohipoor R, Anesi SD, Foster CS (2016). Successful Treatment Strategies in Granulomatosis with Polyangiitis-Associated Peripheral Ulcerative Keratitis. Cornea.

[26] Lu CW, Zhou DD, Wang J, Hao JL (2016). Surgical treatment of peripheral ulcerative keratitis and necrotizing scleritis in granulomatosis with polyangiitis. Saudi Med J.

[27] Bitik B, Kılıç L, Küçükşahin O, Şahin K, Tufan A, Karadağ Ö (2015). Retro-orbital granuloma associated with granulomatosis with polyangiitis: A series of nine cases. Rheumatol Int.

[28] Holle JU, Voigt C, Both M, Holl-Ulrich K, Nolle B, Laudien M (2013). Orbital masses in granulomatosis with polyangiitis are associated with a refractory course and a high burden of local damage. Rheumatology.

[29] Hernandez-Rodriguez J, Hoffman GS, Koening CL (2010). Surgical interventions and local therapy for Wegener’s granulomatosis. Curr Opin Rheumatol.

[30] Ungprasert P, Crowson CS, Cartin-Ceba R, Garrity JA, Smith WM, Specks U (2017). Clinical characteristics of inflammatory ocular disease in anti-neutrophil cytoplasmic antibody associated vasculitis: a retrospective cohort study. Rheumatology.

[31] Sfiniadaki E, Tsiara I, Theodossiadis P, Chatziralli I (2019). Ocular Manifestations of Granulomatosis with Polyangiitis: A Review of the Literature. Ophthalmol Ther.

[32] Kwan ASL, Rose GVE (2000). Lacrimal drainage surgery in Wegener’s granulomatosis. Br J Ophthalmol.

[33] Lee BJ, Nelson CC, Lewis CD, Perry JD (2012). External Dacryocystorhinostomy Surgery in Patients With Wegener Granulomatosis. Ophthal Plast Reconstr Surg.

